# EDLM: Ensemble Deep Learning Model to Detect Mutation for the Early Detection of Cholangiocarcinoma

**DOI:** 10.3390/genes14051104

**Published:** 2023-05-18

**Authors:** Asghar Ali Shah, Fahad Alturise, Tamim Alkhalifah, Amna Faisal, Yaser Daanial Khan

**Affiliations:** 1 Department of Computer Science, Bahria University, Islamabad 04408, Pakistan; aashah.buic@bahria.edu.pk; 2 Department of Computer, College of Science and Arts in Ar Rass, Qassim University, Ar Rass 51921, Qassim, Saudi Arabia; 3 Department of Computer Science, Bahria University, Lahore 54782, Pakistan; amnaf3609@gmail.com; 4 Department of Computer Science, University of Management and Technology, Lahore 54782, Pakistan; yaser.khan@umt.edu.pk

**Keywords:** cholangiocarcinoma (CCA) detection, long short-term memory (LSTM), gated recurrent units (GRUs), bi-directional LSTM (BLSTM), mutation detection, Artificial Intelligence, Machine Learning, Deep Learning, Ensemble learning, Cancer detection, Next generation sequences (NGS)

## Abstract

The most common cause of mortality and disability globally right now is cholangiocarcinoma, one of the worst forms of cancer that may affect people. When cholangiocarcinoma develops, the DNA of the bile duct cells is altered. Cholangiocarcinoma claims the lives of about 7000 individuals annually. Women pass away less often than men. Asians have the greatest fatality rate. Following Whites (20%) and Asians (22%), African Americans (45%) saw the greatest increase in cholangiocarcinoma mortality between 2021 and 2022. For instance, 60–70% of cholangiocarcinoma patients have local infiltration or distant metastases, which makes them unable to receive a curative surgical procedure. Across the board, the median survival time is less than a year. Many researchers work hard to detect cholangiocarcinoma, but this is after the appearance of symptoms, which is late detection. If cholangiocarcinoma progression is detected at an earlier stage, then it will help doctors and patients in treatment. Therefore, an ensemble deep learning model (EDLM), which consists of three deep learning algorithms—long short-term model (LSTM), gated recurrent units (GRUs), and bi-directional LSTM (BLSTM)—is developed for the early identification of cholangiocarcinoma. Several tests are presented, such as a 10-fold cross-validation test (10-FCVT), an independent set test (IST), and a self-consistency test (SCT). Several statistical techniques are used to evaluate the proposed model, such as accuracy (Acc), sensitivity (Sn), specificity (Sp), and Matthew’s correlation coefficient (MCC). There are 672 mutations in 45 distinct cholangiocarcinoma genes among the 516 human samples included in the proposed study. The IST has the highest Acc at 98%, outperforming all other validation approaches.

## 1. Introduction

With the continuous expansion of medical technology, the time of “big data” has arrived. Artificial intelligence (AI) and many AI technologies are being utilized in the medical services industry to unlock the unlimited potential of big data [1]. Cholangiocarcinoma is one of the deadliest forms of cancer in people and is now the number one killer and disability in the world [2]. Cholangiocarcinoma develops when the DNA of bile duct cells is altered. The DNA of a cell conveys guidelines that direct the cell’s activities. Due to these modifications, cells grow uncontrolled and aggregate into masses known as tumors, which can infiltrate and damage healthy bodily parts [3].

The tumor suppressor gene TP53 is mostly responsible for the alterations in cholangiocarcinoma. Additionally, bile duct cancer may be influenced by the genes KRAS, HER2, and ALK. Some of the genetic modifications that lead to bile duct cancer may be influenced by inflammation [3].

**Figure 1:**
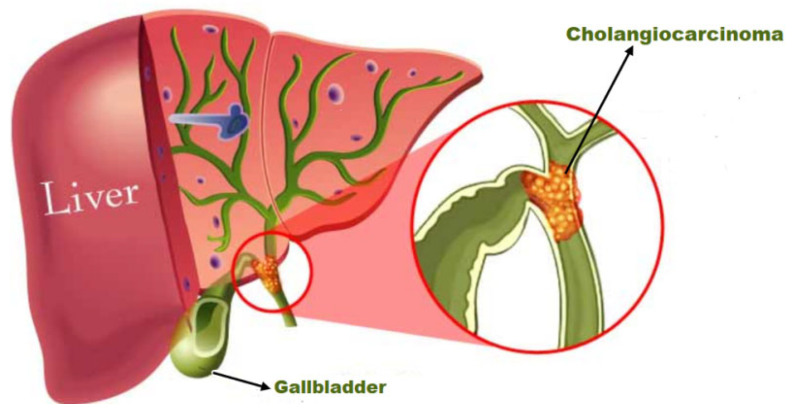
The development of cholangiocarcinoma.

The process of the development of Cholangiocarcinoma is explained with the help of Figure 1. Cholangiocarcinoma is divided into three most common categories: Extrahepatic cholangiocarcinoma is a disease of the extrahepatic bile channels [4]. The cancer may end up in the liver or the small intestine. Cholangiocarcinoma, which begins outside the liver but in the region where the bile ducts and main blood arteries join the liver, is a subclass of extrahepatic cholangiocarcinoma [5]. The second one is intrahepatic cholangiocarcinoma, a cancer of the bile channels in the liver, and the third one is gallbladder cholangiocarcinoma, which starts in the gallbladder.

Cholangiocarcinoma kills about 7000 people a year. Women die less often than men. Asians have the highest death rate. Between 2021 and 2022, African Americans had the largest increase in cholangiocarcinoma deaths (45%), Whites (20%) and Asians (22%) were next [6]. A total of 60–70% of patients with cholangiocarcinoma are diagnosed with local infiltration or distant metastases, thus losing the possibility of a curative surgical intervention. Less than a year is the median survival time across the board [7].

Medical imaging, which includes computed tomography (CT), magnetic resonance imaging (MRI), and ultrasound, is the most effective and common non-invasive diagnostic method for cholangiocarcinoma identification (US) [8]. Cholangiocarcinoma diagnosis could be aided by artificial intelligence (AI). Given the rarity of the condition, the heterogeneity of the tumor’s anatomic location and risk factors, and the importance of AI in cholangiocarcinoma detection [9], several machine learning (ML) AI methods, such as logistic regression, support vector machines (SVMs), artificial neural networks (ANNs), and convolutional neural networks (CNNs), have been used to identify cholangiocarcinoma [10]. This study uses a machine learning framework to pinpoint cholangiocarcinoma.

A rare kind of cancer called cholangiocarcinoma starts in the bile ducts. The small intestine receives bile (a digestive liquid) from the liver and gallbladder through bile ducts, which are tiny tubes. Most cholangiocarcinoma cases are discovered after the disease has progressed outside the bile ducts [11]. Treatment is tough, and the chances of recovery are often slim. The exact etiology of cholangiocarcinoma is not known by experts. Risk factors suggest that diseases that cause chronic (permanent) irritation of the bile ducts may contribute to the growth of this cancer [12]. DNA changes resulting from constant damage, such as inflammation, can alter some cells’ growth, division, and behavior. The following pie chart shows that more than 70% of liver tumors are caused by hepatocellular carcinoma (HCC), and 8% of liver cancers are cholangiocarcinomas (CCAs), the second most common primary malignancy [13].

**Figure 2:**
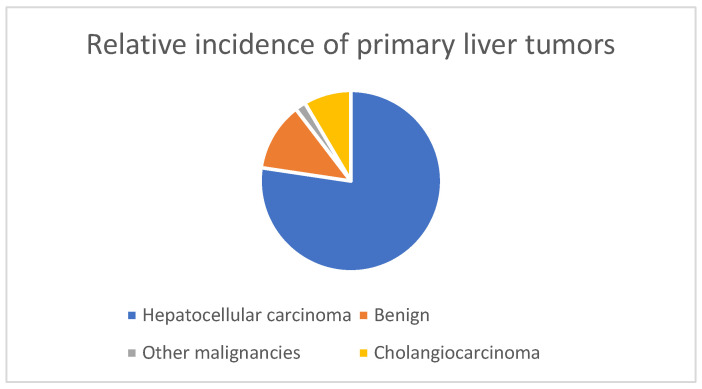
The relative incidence of primary liver tumors.

The ratio of primary tumors is shown in Figure 2. Most patients with cholangiocarcinoma are over 65 years old. Effective treatment can be difficult because the disease is often not diagnosed until it is at an advanced stage. Depending on where the cancer is and how it develops, affected people can live months to years after diagnosis [13]. In medicine, many machine learning and deep learning technologies are used to detect and prevent various diseases. An artificial neural network (ANN) was demonstrated by Matake et al. in 2006, which can distinguish between the four liver masses (HCC, CCA, hemangioma, and metastasis), and reported an average AUC of 0.961 [14]. 

In 2009, Logeswaran used a popular ANN, a multilayer perceptron, to differentiate images with and without CCA [15]. As a result, the Acc of the test for distinguishing healthy images from tumor images was 94%, and the Acc of the multi-disease test was 88%. To assess the Sp of several serum indicators to enhance CCA diagnosis, Pattanapairoj et al. constructed a classification model in 2015 using both C4.5 (the technique used to build classification models for decision trees in logical form) and ANN, with an AUC of 0.961 [16]. Shao et al. created an ANN model in 2018, which is crucial for patients with inoperable CCA when choosing a course of therapy, with an AUC of 0.964 [17].

Another study by Peng et al., in 2019, offered a novel method of precision treatment for CCA patients, using radiographic signatures of 128 CCA patients based on US scans to noninvasively characterize the biological activity of CCA, with an AUC of 0.930 [18] as shown Table 1. Additionally, Yang et al. investigated the MRI radiomics model’s diagnostic efficacy using random forests in 2020, reporting an AUC of 0.90 [19].

The AUC values were the best results in the above studies. ANN—artificial neural network; MLP—multi-layer perceptron; C4.5—an algorithm used to construct a decision tree classification model in logical form; BP-ANN—back propagation artificial neural network; LASSO—least absolute shrinkage and selection operator; and SVM—support vector machine [13].

Artificial intelligence can automatically offer a quantitative and impartial evaluation of a tumor by recognizing intricate patterns in picture data [20]. The present status of the artwork has a great deal of limitations and cutoff points. There is not yet a clear benchmark dataset for cholangiocarcinoma transformations and specific successions. Additionally, assessment techniques come up short on essential thoroughness and conviction. Subsequently, apparently, there is sufficient space for the models’ precision to be moved along [21]. The latest and most summed-up dataset, as portrayed in the data acquisition framework section, was collected for this study while keeping these requirements in mind. Three deep learning RNN algorithms were utilized in this study: LSTM, GRU, and BLSTM.

Many machine and deep learning algorithms have been utilized for cancer detection, primarily focusing on image-based approaches that identify cancer after symptoms have already appeared. However, early detection is crucial to enhance treatment outcomes. This study aims to achieve early detection by detecting cancer through mutation detection in gene sequences. The dataset used in this study is not publicly available as it is formulated from multiple renowned databases in this study. To enable efficient mutation detection, extensive feature extraction techniques are employed. Moreover, the training process is enhanced by ensemble multiple deep learning algorithms. The performance of the proposed models is evaluated using various testing techniques to ensure their effectiveness.

## 2. Materials and Methods

**Figure 3:**
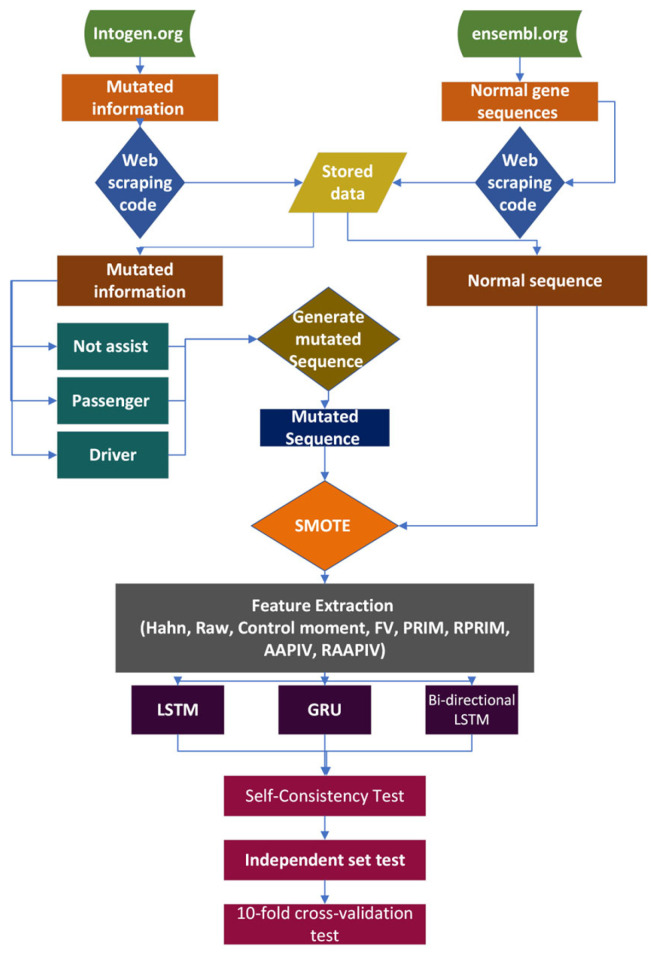
Methodology of the proposed framework for the detection of cholangiocarcinoma.

This section talks about the inside activity and full clarification of the dataset collection, feature extraction, and classification strategies. The proposed methodology consists of dataset curation, extensive feature extraction, EDLM for accurate classification, testing, and evaluation. The proposed EDLM consists of three deep learning models—LSTM, GRU, and BLSTM. The whole process is explained with the help of Figure 3. 

### 2.1. Data Acquisition Framework

The dataset is the main part of this study. A complete data collection framework was built to train, test, and evaluate the EDLM. The most common way of obtaining reliable and exact information for a study is known as information assortment. Information procurement is the most common way of gathering information to direct research and illustrating how the information is accumulated from a solid source [22].

In human genes, two types of mutations occur: driver mutations, passenger mutations. The type of mutation in cells that causes cancer is known as a driver mutation. Cells grow abnormally because of driver mutation [23]. The dataset was developed so that it contains both normal and mutated sequences. The normal gene sequences were gathered from asia.ensembl.org (accessed on 13 November 2022) [24] using web scrapping code (WSC) developed in Python. CD-HIT was used to reduce sequence redundancy and improve performance. These gene sequences are available, but a generalized dataset related to mutated sequences is not available. Therefore, mutation information was obtained from IntOGen.org [25] using WSC written in Python. The mutation information contains the address of each element in the normal gene sequence. It has both nucleotides before mutation and after mutation. Therefore, a code was written in Python named Generated Mutated sequences (GMSs). which incorporated these changes in the normal gene sequences and built mutated sequences. All the normal gene sequences of all genes were combined in one file, and all the mutated gene sequences of all genes were combined in another file. Thus, the final dataset was formulated to have both normal gene sequences and mutated gene sequences. 

The 516 human samples included in the proposed study contain 672 mutations related to 45 different cholangiocarcinoma genes. In Table 2, cholangiocarcinoma genes are listed.

**Figure 4:**
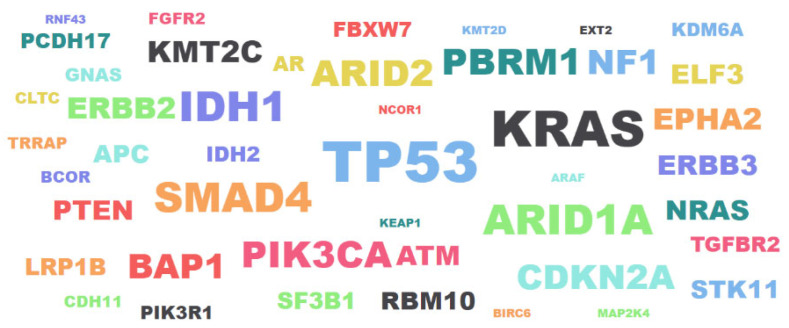
Word cloud of cholangiocarcinoma genes.

A word cloud visualization generated with Python to highlight the nucleotides and the frequency and significance of each nucleotide in all gene sequences connected to cholangiocarcinoma are represented with the size of each gene in Figure 4.

### 2.2. Feature Extraction

Feature extraction effectively reduces the amount of data that must be processed while accurately and comprehensively defining the initial data set by combining variables with selections and/or characteristics. It enhances the performance and Acc of training models [26].

Feature extraction techniques are used to extract key characteristics from the raw data source. The process of collecting data in numerous phases to extract critical characteristics required in model training is known as feature extraction [27]. This is the most crucial phase in the preparation of machine learning and deep learning algorithms. Attribute extraction finds data patterns, which are then employed in data training and testing procedures. 

**Figure 5:**
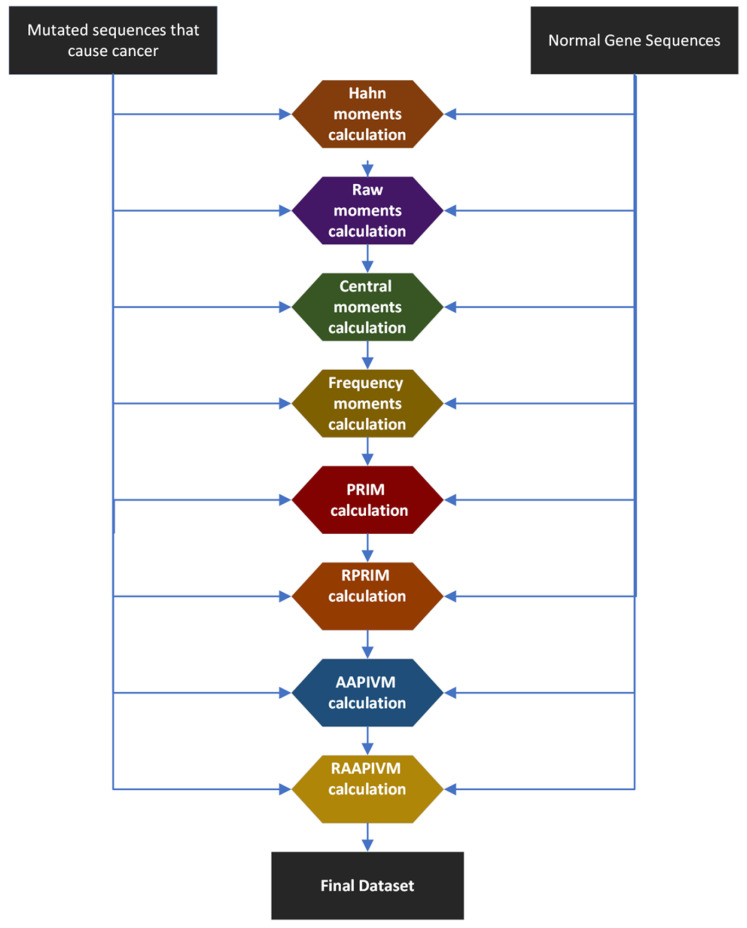
Extensive feature extraction techniques for the cholangiocarcinoma dataset.

This study calculates statistical moments such as Hahn, raw, and central moments. These feature extraction approaches are used to extract key features from data from mutant gene sequences and normal gene sequences [27,28]. All feature extraction techniques are listed in Figure 5.

#### 2.2.1. Hahn Moments Calculation

Hahn moments are used to compute statistical parameters. Hahn moments are the most important concept in pattern recognition. They compute the mean and variance for the data collection. The Hahn polynomial is used to calculate Hahn moments. The size and placement of these Hahn points remain constant. These details are significant because they are sensitive to biological sequence information and can extract hidden properties from gene sequences [29].

Hahn moments need the use of two-dimensional data. As a result, the genomic sequences are transformed into a two-dimensional matrix $A^{\prime }$ of size x∗x, as shown in the following equation:

$$A^{\prime }=\left[\begin{array}{ccc} A_{11} & A_{12} & A_{1n}\\ A_{21} & A_{22} & A_{2n}\\ \vdots & \vdots & \vdots \\ A_{n1} & A_{n2} & A_{nn} \end{array}\right]$$

The gene sequence is defined by *A*′ in this case. The value of *A*′ is used to compute Hahn moments.

Each element *A*′ represents a genomic sequence residue. The computation of statistical moments of the third order can be found in [30]. Hahn moments are orthogonal since they take in a square matrix. For the benchmark dataset, the Hahn polynomial can be calculated using the following equations:

$$h_{m}^{a,b}(C,D)=(D+V-1{)}_{m}(D-1{)}_{m}\sum_{z=0}^{m}\;(-1{)}^{z}\frac{(-m{)}_{z}(-C{)}_{z}(2D+a+b-m-1{)}_{z}}{{\left.\left(D+b-1{)}_{z}(D-1\right)\right)}_{z}}\frac{1}{z!}$$

All integers a and b must be positive and are predefined constants. The size of the data array is G, and the instant’s order is

$$A_{p,q}=\sum_{j=0}^{G-1}\;\sum_{i=0}^{G-1}\;\delta_{p,q}h_{p}^{a,b}(j,G)h_{q}^{a,b}(j,G),m,n=0,1,2,\ldots ,G-1$$

where a,b are predetermined constants, is any member of the square matrix and represents the current order.

If A is an integer, then [0,G−1](G is the provided positive integer). The polynomial’s shape can be altered using these moveable variables [31]. Pochhammer’s symbol is $(a)k=a\cdot (a+1)\cdots (a+k-1)=\frac{r(a+k)}{r(a)}$. Equations (2) and (3) may be used to easily determine the normalized Hahn moments of any order. The following numbers stand for the Hahn-moment-based distinctive features: $H_{00},H_{01},H_{10},H_{11},H_{02},H_{12},H_{21},H_{30},H_{03}.$ For each gene sequence, 10 raw, 10 central, and 10 Hahn moments are calculated. These moments are united into the miscellaneous super feature vectors and are up to 3rd order.

#### 2.2.2. Central Moments Calculation

Utilizing mean and variance, the central moment of feature extraction is utilized to extract key features. It is the region closest to the mean of the randomly chosen variable in the probability distribution [32]. The following equation illustrates the generic formula for calculating the central moments for the cholangiocarcinoma dataset:

$$C_{a,b}=\sum_{g=1}^{m}\;\sum_{h=1}^{m}\;(e-x{)}^{a}(h-y{)}^{b}\delta_{gh}$$

$C_{00},C_{01},C_{10},C_{11},C_{02},C_{12},C_{21},C_{30},\;\mathrm{and}\;C_{03}$ are the designations for the distinctive qualities from central moments up to the 3rd order.

#### 2.2.3. Raw Moments Calculation

The raw moments are used for statistical computations. Imputation is the process of maintaining facts by substituting the best available replacement values for missing data values in a data collection. The following equation shows the initial moments for the 2D data of order x+y [33]:

$$R_{x,y}=\sum_{g=1}^{m}\;\sum_{h=1}^{m}\;g^{x}h^{y}\delta_{gh}$$

Up to order three, raw moments are computed. This provides detailed information on important sequence elements, including $R_{00},R_{01},R_{10},R_{02},R_{20},R_{03},\;\mathrm{and}\;R_{30}$.

#### 2.2.4. Position Relative Incidence Matrix (PRIM)

The location of each gene in the cholangiocarcinoma gene sequence is determined using PRIM [31]. The following equation displays a PRIM-generated matrix with a dimension of 30 × 30:

$$P_{PRIM}=\left[\begin{array}{cccccc} P_{1\to 1} & P_{1\to 2} & \cdots & P_{1\to j} & \cdots & P_{1\to m}\\ P_{2\to 1} & P_{2\to 2} & \cdots & P_{2\to j} & \cdots & P_{2\to m}\\ \vdots & \vdots & & \vdots & & \vdots \\ P_{n\to 1} & P_{n\to 2} & \cdots & P_{n\to j} & \cdots & P_{n\to m}\\ \vdots & \vdots & & \vdots & & \vdots \\ P_{m\to 1} & P_{m\to 2} & \cdots & P_{m\to j} & \cdots & P_{m\to m} \end{array}\right]$$

#### 2.2.5. Reverse Position Relative Incidence Matrix (RPRIM)

The RPRIM operates similarly to PRIM but in the other direction [31]. The following equation describes, in detail, how to calculate RPRIM for the cholangiocarcinoma dataset:

$$R_{PRIM}=\left[\begin{array}{cccccc} R_{1\to 1} & R_{1\to 2} & \cdots & R_{1\to j} & \cdots & R_{1\to m}\\ R_{2\to 1} & R_{2\to 2} & \cdots & R_{2\to j} & \cdots & R_{2\to m}\\ \vdots & \vdots & & \vdots & & \vdots \\ R_{n\to 1} & R_{n\to 2} & \cdots & R_{n\to j} & \cdots & R_{n\to m}\\ \vdots & \vdots & & \vdots & & \vdots \\ R_{m\to 1} & R_{m\to 2} & \cdots & R_{m\to j} & \cdots & R_{m\to m} \end{array}\right]$$

#### 2.2.6. Accumulative Absolute Position Incidence Vector (AAPIV)

A frequency matrix provides information about the frequency of a gene in a sequence of genes. AAPIV describes the different nucleotide configurations that occur in gene sequences. AAPIV is used to study the nucleotide sequences of cancer gene sequences against each other. The following equation serves as an illustration of the relative gene sequences of cholangiocarcinoma [33]:

$$K=\left\{\lambda_{1},\lambda_{2},\ldots ,\lambda_{n}\right\}$$

where n is the number of total nucleotides in the gene sequence, which may be estimated using equation regardless of its component:

$$\lambda i=\sum_{j=1}^{m}\;\beta_{j}$$

where βj represents where the ith nucleotide is located.

#### 2.2.7. Reverse Accumulative Absolute Position Incidence Vector (RAAPIV)

RAAPIV functions similarly to AAPIV but in the other direction. Here, the RAAPIV equation is presented:

$$\lambda =\left\{n_{1},n_{2},\ldots ,n_{m}\right\}$$

#### 2.2.8. Frequency Vector Calculation

A dataset consists of tens of thousands of data records, each with a unique set of properties. The sequence of genes that come together to produce a gene sequence is represented by a frequency matrix. This is represented by the following equation:

$$\alpha =\left\{\varepsilon_{1},\varepsilon_{2},\ldots ,\varepsilon_{n}\right\}$$

The frequency of each gene in the cholangiocarcinoma gene sequence may be seen below. The following equation can be used to determine the frequency vector:

$$FV=\left\{f_{1},f_{2},\ldots ,f_{n}\right\}$$

The frequency of each gene in the gene sequence is shown here by the numbers f_1_ to f_n_.

## 3. Proposed Deep Learning Algorithms 

In this study, an ensemble deep learning model (EDLM) is proposed, which consists of three deep learning models—LSTM, GRU, and BLSTM—for the early diagnosis of cholangiocarcinoma. Several malignancies, including cholangiocarcinoma, are recognized, detected, predicted, and diagnosed using the proposed EDLM. An input layer, an output layer, a pooling layer, a dense layer, and a dropout layer are just a few of the layers that make up a deep neural network model. Fully connected layers are then placed on top of everything else [34]. Each layer receives input from the layer before it and analyses the features. Algorithms with intrinsic learning characteristics inside these layers can educate themselves using several learning techniques.

LSTM, GRU, and BLSTM are three types of deep learning RNN algorithms [35] used in this study. For the detection of cholangiocarcinoma, these algorithms employ three assessment methods: An SCT, IST, and 10-FCVT.

### 3.1. Long Short-Term Memory (LSTM)

**Figure 6:**
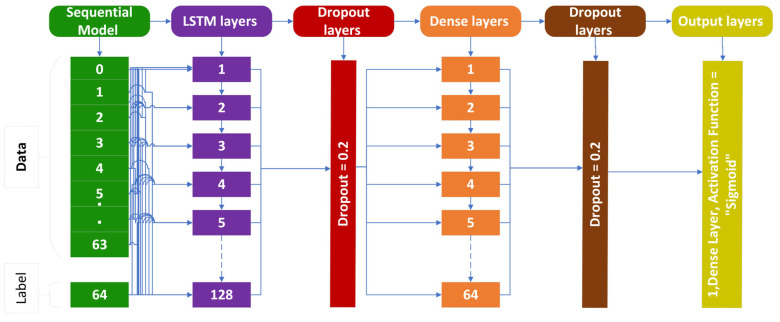
LSTM cell structure for cholangiocarcinoma.

In challenges involving sequence prediction, LSTM networks, a kind of recurrent neural network, may discover order dependency [36]. The efficiency of the RNN declines with slot length. In essence, LSTMs are capable of long-term information storage. The information’s length is modified to 64. The addition of a 128-neuron LSTM layer is also mentioned in [37]. The dense layer links the feedback from all the layers and transmits it to the result layer. A 20% dropout capacity is added in the dropout layer to prevent the model from overfitting. In order to combat model overfitting, this model features two dropout layers. In the LSTM layer, Stochastic Gradient Descent (SGD) is utilized as an enhancer. A sigmoid capability is utilized as an initiation capability, the details of which are also available in [38]. These details are also shown with the help of Figure 6.

Which input value is used to change the memory depends on the input gate. Whether 0 or 1 values are allowed is determined by the sigmoid function [39]. The tanh function also assigns a weight to the provided data, defining its value on a scale from −1 to 1.

$$\begin{array}{c} i_{t}=\sigma \left(W_{i}\cdot \left[h_{t}-1,x_{t}\right]+bi\right)\\ c_{t}=tanh\left(W_{c}\cdot \left[h_{t}-1,x_{t}\right]+bc\right) \end{array}$$

The Forget gate identifies the details in the block that should be erased. A sigmoid function determines it. It examines the previous state (h_t_ − 1) and the content input (x_t_) for each number in the cell state, c_t_ − 1, and returns a value between 0 (omit this) and 1 [39].

$$f_{t}=\sigma \left(W_{f.}\left[h_{t}-1,x_{t}\right]+bf\right)$$

The block’s input and memory are both used to calculate the output. This is determined by the sigmoid function whether 0 or 1 values are acceptable. Additionally, the tanh function defines which values between 0 and 1 can pass [40]. Additionally, the tanh function gives the provided values weight by determining their significance on a scale from −1 to 1 and multiplying it by the sigmoid output.

$$\begin{array}{c} o_{t}=\sigma \left(W_{o}\cdot \left[h_{t}-1,x_{t}\right]+bo\right)\\ h_{t}=o_{t}*tanh\left(c_{t}\right) \end{array}$$

### 3.2. Gated Recurrent Unit (GRU)

**Figure 7:**
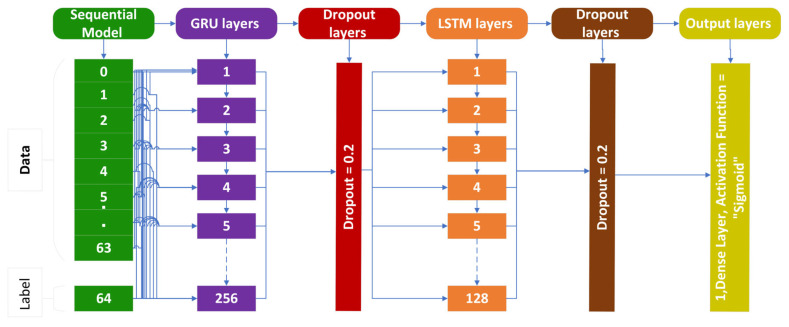
GRU cell structure for cholangiocarcinoma.

The second deep learning technique used in the suggested study is the GRU strategy. GRU accomplishes comparable tasks as LSTM but has fewer gates. GRU outperforms LSTM in terms of outcomes [40] since it uses fewer gates and parameters. The reset gate and the update gate are the only gates used by GRU in the cell. The update gate in the GRU controls how much past data are used, whereas the reset gate controls how much past data are disregarded [41]. The GRU cell structure utilized to identify cholangiocarcinomas is shown in the Figure 7.

The following equations show the work process of GRU:

$$\begin{array}{c} r_{t}=\sigma \left(x_{t}U^{r}+B_{t-1}W^{r}\right)\\ z_{t}=\sigma \left(x_{t}U^{z}+B_{t-1}W^{z}\right)\\ h_{t}^{\prime }=tanh\left(r_{t}*B_{t-1}U+x_{t}W\right)\\ \left.y_{t}=\left(1-z_{t}\right)*B_{t}^{\prime }+z_{t}*B_{t-1}\right) \end{array}$$

In the proposed model, the input is transformed into a vector with a fixed word length of 64 by a single embedding layer. A GRU layer with 256 neurons and a fundamental RNN layer with 128 neurons make up the second layer. Two dropout layers are added at 30% to avoid overfitting. A substantial layer of 10 neurons is introduced at the end. Stochastic Gradient Descent (SGD) is used as an optimizer in the GRU layer, as explained in [42]. As an activation function, the sigmoid function is used. Sparse categorical cross entropy (SCCE) is used to reduce the loss experienced during training the proposed model.

### 3.3. Bi-Directional LSTM (BLSTM)

**Figure 8:**
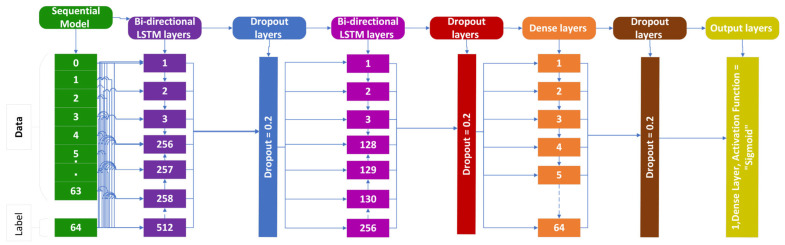
BLSTM cell structure for cholangiocarcinoma.

The BLSTM extends the regular LSTM. The model incorporates two parallel LSTM layers to produce a forward and backward loop, as seen in the picture below [42]. The network is supposed to produce predictions by using past and future information from forward and backward sequences. In this scenario, the present information is dependent on previous information and is also related to future information [43]. The red and green arrows in the Figure 8 and the equations below reflect the forward and backward consequences, respectively.

$$\begin{array}{c} h_{t}^{\to }=g(U_{h\to }x_{t}+W_{h\to }\vec{h_{t-1}}+b_{h}\to )\\ h_{t}^{\leftarrow }=g\left(U_{h}\leftarrow x_{t}+W_{h}\leftarrow h_{t-1}^{\leftarrow }+b_{h}\leftarrow \right)\\ y_{t}=g\left(V_{h}\to h_{t}\to +V_{h}\leftarrow h_{t}^{\leftarrow }+b_{y}\right) \end{array}$$

The following Figure 8 explains the BLSTM cell structure used in the identification of cholangiocarcinoma [44,45].

## 4. Ensemble Deep Learning Models (EDLM) 

EDLM is a cycle where different assorted models are made to foresee a result, either by utilizing an assortment of modeling algorithms or by utilizing an assortment of training data sets. The model then, at that point, joins each base model’s conjecture, yielding a solitary last expectation for the concealed information. The purpose of using EDLM is to reduce prediction generalization errors. When using the EDLM, the prediction error of a model that is different and independent from the base model is reduced. Technology seeks public wisdom when making predictions. An ensemble model has multiple base models, but it works and functions as a single model [46].

**Figure 9:**
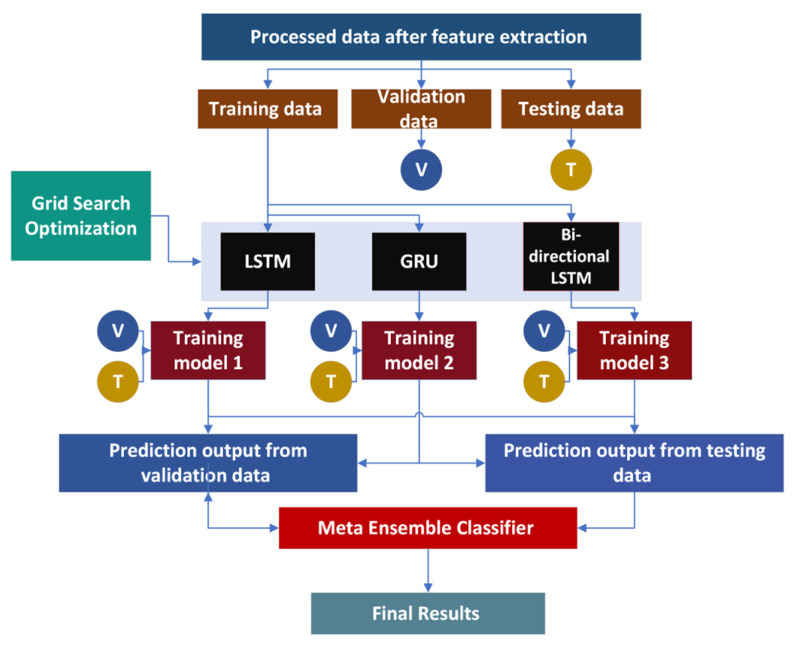
The process of proposed EDLM.

With the use of a group learning strategy, this study focuses on the demonstration of unique deep learning models, including LSTM, GRU, and BLSTM. Three groups, such as the training set, validation set, and test set, are created from the extensive feature-extracted dataset. V denotes the validation set, whereas T denotes the test set [47]. Every single LSTM, GRU, and BLSTM deep learning model receives the training set as a contribution. In order to get scan ranges and the optimal attributes for suggested ensemble learning model bounds, the matrix inquiry improvement approach is also used. As shown in Figure 9, EDLM is created from each unique deep learning model under the names training model1, training model2, and training model3; these models are represented as LSTM, GRU, and BLSTM, respectively. Similar studies have also been conducted using machine and deep learning techniques [47,48,49,50,51,52,53,54,55,56,57,58,59,60,61]. 

Testing and validation sets are utilized to assess each training model. At last, as displayed in the outcomes area, an EDLM produces last superior outcomes.

$$gp,i=\sum_{n=1}^{N}\;w_{n}f_{n},i$$

The singular profound learning model is provided loads to fabricate group learning forecast in the situation. Here, n signifies the weight given to every exceptional profound learning model, means each model’s forecast, and is the perception [62].

The numerical recipes used to decide the results of the calculations are recorded below. The recipes to determine the responsiveness, particularity, exactness, and Matthew’s correlation coefficient (MCC) are portrayed in the equations below, in a specific order:

$$\begin{array}{c} Sn=TP/(TP+FN)\\ Sp=TN/(TN+FP)\\ Acc=(TP+TN)/(TP+FP+FN+TN) \end{array}$$

$$MCC=\frac{\left(TPXTN)-(FPXFN\right)}{\sqrt{\left(TP+FP)(TP+FN)(TN+FP)(TN+FN\right)}}$$

In these equations, TN,TP,FN,and FP are represented by every true negative value, all the dataset’s true positive values, false negative values, and false positive values, respectively. 

Sn in the equations above relates to the capacity to forecast the count that can accurately detect cholangiocarcinoma. The capacity to anticipate the count that will really reveal the lack of cholangiocarcinoma is referred to as Sp. All individuals in TP + FN have the specified condition [63], while TN + FP are the subjects devoid of the mentioned circumstances. Total participants with good outcomes are denoted by TP + FP, whereas patients with negative results are denoted by TN + FN [64,65]. 

## 5. Results

To extract the key aspects of the balanced data, the cholangiocarcinoma dataset is pre-processed. To the retrieved data, extensive feature extraction techniques are applied. Then, the proposed EDLM deep learning methods are employed. IST, SCT, and 10-FCVT were used to gauge the effectiveness of the deep learning algorithm. The outcomes of various validation procedures are as follows.

### 5.1. Self-Consistency Test (SCT)

**Figure 10:**
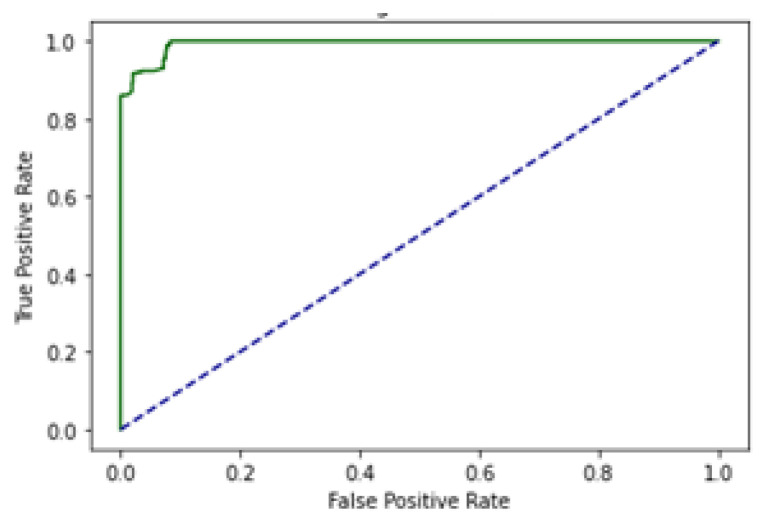
ROC curve of EDLM in SCT.

SCT is an iterative testing process; when results are satisfied, it stops. A total of 100% of the information collected during an SCT is used for testing and training. The complete dataset is used in SCT for both training and testing. Very minimal loss occurs in BLSTM. In contrast, the SCT showed that LSTM, GRU, and BLSTM had extremely excellent Acc. The ROC curve using SCT is shown in Figure 10. 

**Figure 11:**
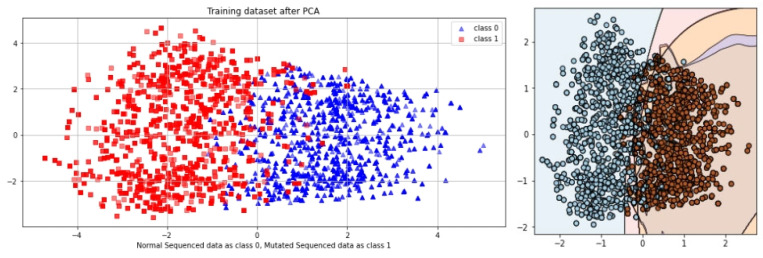
Decision boundary result of EDLM in SCT.

The outcome, defined by the ROC curve as being between 0.99 and 1.0, should be regarded as excellent. The decision boundary results using SCT are shown in Figure 11.

### 5.2. Independent Set Test (IST)

**Figure 12:**
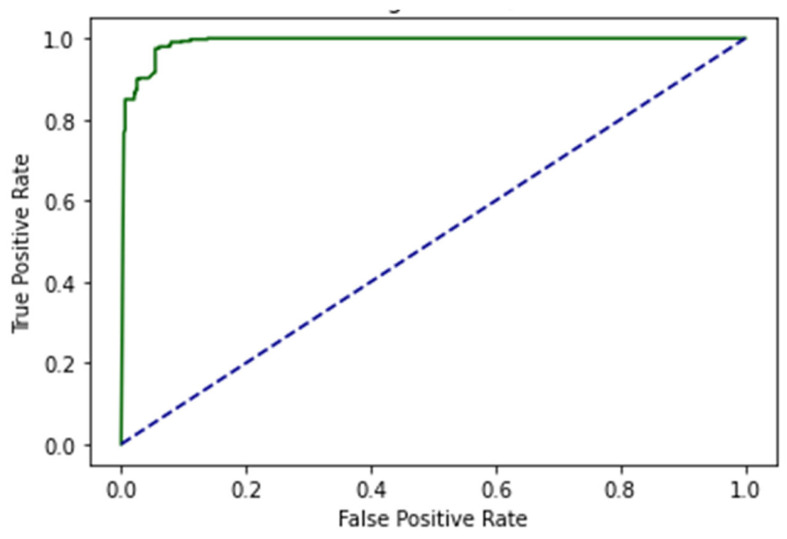
ROC curve of EDLM in IST.

The proposed study was also validated via IST. The values from the confusion matrix are used to determine the model’s Acc. This test is related to the primary performance measuring method for the proposed model. In this case, the algorithm is trained on 80% of the dataset and tested on 20% of the dataset. The ROC curve of EDLM in IST is shown in Figure 12.

**Figure 13:**
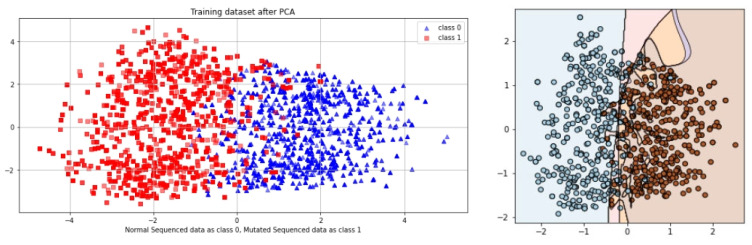
Decision boundary result of EDLM in IST.

The decision boundary result using IST are shown in Figure 13.

### 5.3. 10-Fold Cross-Validation (10-FCVT)

**Figure 14:**
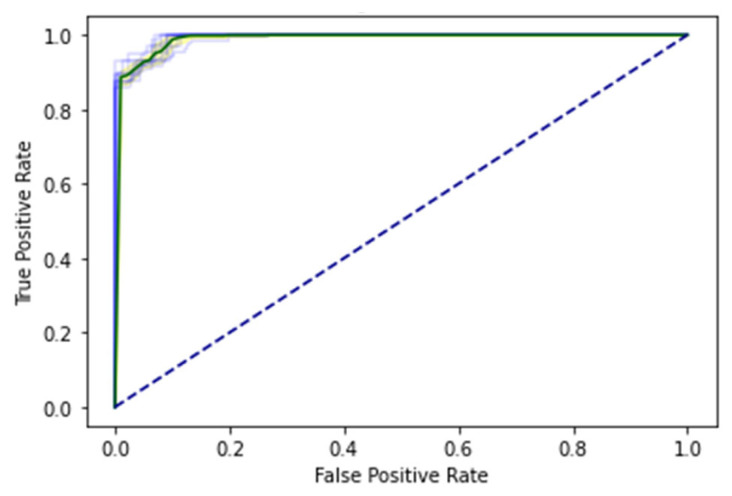
ROC curve of 10-fold c-val.

The data are uniformly subsampled into 10 groups for the 10-FCVT. By dividing the training set into 10 divisions, treating each part in the validation set, and training the rest 9-fold, you may choose the model’s hyperparameters and architecture. This process is repeated 10 times, and then, the average value is calculated. Figure 14 shows the ROC curve of EDLM in 10-FCVT.

**Figure 15:**
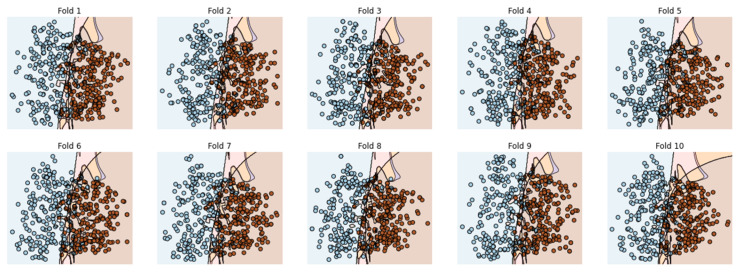
Decision boundary of EDLM in 10-FCVT.

A decision boundary visualization of each fold obtained through EDLM in 10-FCVT is shown in Figure 15.

### 5.4. Results Comparison 

The result of the EDLM is contrasted with those of its own distinctive algorithms, including LSTM, GRU, and BLSTM, in Table 3. Many measurements are used for comparison. A comparison is made using all three types of tests, such as SCT, IST, and 10-FCVT. Table 3 demonstrates how the proposed EDLM improves the identification precision of the stand-alone deep learning techniques, including LSTM, GRU, and BLSTM. In Table 3, it is clearly mentioned that BLSTM performs very well in SCT and IST, and on the other side, EDLM performs very well in 10-FCVT, which is the best representation of the whole dataset. The statistical tools that evaluate the models are Acc, Sn, Sp, and MCC. The Acc, Sn, Sp, and MCC of BLSTM in SCT are 99%, 100%, 98%, and 0.98, respectively. The Acc, Sn, Sp, and MCC of BLSTM in IST are 98%, 100%, 96%, and 0.95, respectively. The Acc, Sn, Sp, and MCC of EDLM in 10-FCVT are 92%, 94%, 93%, and 0.86, respectively.

Table 4 provides a comprehensive overview of the performance comparison between the previous approaches and proposed models. The BLSTM model stands out with its exceptional AUC value of 0.99, indicating its superior predictive capabilities. Additionally, the proposed algorithms, as a whole, exhibit better accuracies at 98% than those achieved by previous methods, further emphasizing their potential for enhancing predictive modeling tasks. These findings highlight the importance of continued research and development in the field, as advancements in machine learning algorithms have the potential to revolutionize various domains.

The proposed ELDM can also be applied to other types of cancerous datasets. The results produced by the proposed model on a prostate cancer dataset are shown in Table 5. It seems that the proposed model is very effective for the detection of mutations to detect cancer. The model is also validated on other types of cancer datasets, and therefore, this demonstrates the generalizability of the proposed model. 

## 6. Discussion 

Cholangiocarcinoma (CCA), one of the deadliest types of cancer in humans, is currently the leading cause of death and disability worldwide. In this study, an EDLM composed of three deep learning models—LSTM, GRU and BLSTM—is proposed. The proposed system is a viable in silico strategy for tracking down mutations in cholangiocarcinoma. When contrasted with the present status of art, the recommended system is a computationally clever indicator. A complete data collection framework is developed in Python to scrap the data from well-known databases and develop mutated gene sequences. An extensive feature extraction framework is developed to extract useful features from the gene sequences and prepare the dataset for training and testing. The EDLM based on ensemble learning of LSTM, GRU, and BLSTM is developed to learn the hidden features of the prepared dataset and identify mutations in cholangiocarcinoma for early detection. Multiple testing techniques, such as SCT, IST, and 10-FCVT, are used to test the proposed model. Multiple statistical tools, such as Acc, Sn, Sp, and MCC, are used for the evaluation of the proposed EDLM, LSTM, GRU, and BLSTM. The performance of EDLM in terms of ROC curve in SCT, IST, and 10-FCVT is shown with the help of Figure 10, Figure 12 and Figure 14 respectively. The performance of EDLM in terms of decision boundary in SCT, IST, and 10-FCVT is shown with the help of Figure 11, Figure 13 and Figure 15 respectively. The evaluation results are shown in Table 3. The best performance in SCT is shown by BLSTM. The best performance in IST is also shown by BLSTM. The best performance in 10-FCVT is shown by EDLM, as discussed in Table 3. 

## 7. Conclusions

Cholangiocarcinoma (CCA), one of the deadliest types of cancer in humans, is currently the leading cause of death and disability worldwide. The best results for early cholangiocarcinoma cancer diagnosis using EDLM are shown in this study. The proposed EDLM consists of three different deep learning algorithms—LSTM, GRU, and BLSTM— that are used to discover mutations in cholangiocarcinoma. All algorithms have above 95% Acc, as shown in Table 3, with an AUC value of 99. The diagnosis of cholangiocarcinoma made using these results is the most precise to date. Table 3 shows the results of the IST, SCT, and 10-FCVT in terms of Acc, Sn, Sp, and MCC. Right now, these are the best techniques for cholangiocarcinoma early diagnosis. Future studies will build on this effort to identify other diseases, and an ensemble deep learning model of other types will also be made.

## Figures and Tables

**Table 1:** The following table shows a compilation of prior work.

Author	Methods	Area Under the Curve
Matake, et al. [14]	ANN	0.961
Logeswaran [15]	MLP	0.960
Pattanapairoj, et al. [16]	C4.5, ANN	0.961
Shao, et al. [17]	BP-ANN	0.9648
Peng, et al. [18]	LASSO, SVM	0.930
Yang, et al. [19]	Random Forest	0.90

**Table 2:** Genes involved in cholangiocarcinoma and mutations.

Symbol	Mutations	Samples	Symbol	Mutations	Samples
TP53	98	96	SF3B1	13	6
KRAS	65	61	LRP1B	26	6
ARID1A	28	30	IDH2	5	5
SMAD4	24	28	TGFBR2	5	5
IDH1	28	26	AR	9	5
PIK3CA	21	19	PCDH17	6	5
ARID2	21	18	FBXW7	8	5
PBRM1	19	18	GNAS	11	4
BAP1	26	16	KDM6A	6	4
CDKN2A	15	15	PIK3R1	5	4
NF1	17	14	CDH11	8	3
KMT2C	17	11	TRRAP	7	3
ERBB2	13	10	BCOR	5	3
EPHA2	16	10	FGFR2	8	3
ERBB3	14	9	CLTC	8	3
ATM	18	9	KEAP1	1	2
ELF3	9	9	NCOR1	2	2
NRAS	10	8	ARAF	3	2
PTEN	9	8	KMT2D	16	2
APC	13	7	EXT2	3	2
STK11	9	7	MAP2K4	2	2
RBM10	12	7	BIRC6	11	2
RNF43	2	2			

**Table 3:** Comparison of results of evaluation tools of LSTM, GRU, BLSTM, and EDLM.

	Self-Consistency Test (SCT)				Independent Set Test (IST)				10-Fold Cross-Validation Test (10-FCVT)			
	**Acc**	**Sn**	**Sp**	**MCC**	**Acc**	**Sn**	**Sp**	**MCC**	**Acc**	**Sn**	**Sp**	**MCC**
**LSTM**	94%	97%	93%	0.89	93%	95%	91%	0.87	92%	91%	93%	0.85
**GRU**	94%	95%	93%	0.88	94%	96%	92%	0.89	92%	91%	93%	0.84
**BLSTM**	99%	100%	98%	0.98	98%	100%	96%	0.95	92%	91%	93%	0.84
**EDLM**	94%	95%	93%	0.88	96%	98%	94%	0.92	92%	94%	93%	0.86

**Table 4:** Comparison of results of proposed models to other previous results.

Previous Results		
Author	Models	Area Under the Curve
Matake, et al. [14]	ANN	0.961
Logeswaran [15]	MLP	0.960
Pattanapairoj, et al. [16]	C4.5, ANN	0.961
Shao, et al. [17]	BP-ANN	0.9648
Peng, et al. [18]	LASSO, SVM	0.930
Yang, et al. [19]	Random Forest	0.90
**Proposed Results**		
	**Models**	**Area Under the Curve**
	LSTM	0.98
	GRU	0.97
	BLSTM	0.99
	EDLM	0.98

**Table 5:** The results of the proposed ELDM on prostate cancer.

Evaluation Matrices	Values	Evaluation Matrices	Values
Accuracy	98.87%	Precision	98.87%
Sensitivity	99.50%	Recall	98.87%
Specificity	99.63%	F1 Score	98.87%
MCC	0.98	AUC	0.99
